# Rare presentation of urachal remnant with suprapubic sinus: A case report

**DOI:** 10.1016/j.eucr.2025.102962

**Published:** 2025-01-29

**Authors:** Sarah Cairo, Gabriella Grisotti, Hanmin Lee

**Affiliations:** University of California San Francisco, Division of Pediatric Surgery, USA

**Keywords:** Urachal remnant, Urachal abscess, Pediatric surgery, Congenital anomalies, Vitelline duct remnant

## Abstract

Incomplete obliteration of the allantois during development gives rise to urachal remnants. Presentation is variable and can range from draining umbilicus to chronic granuloma to acute infection requiring management with antibiotics and surgical excision. High index of suspicion is required to diagnoses more unusual presentations. This is a report on a case of urachal remnant presenting as a suprapubic sinus in a pediatric patient.

## Highlights

• Urachal remnants can have variable presentations in pediatric patients.
• Presentation and diagnosis are often delayed and require a high index of suspicion.
• Staged approach to excision of infected urachal remnants is associated with good outcomes.

## 1 Introduction

Urachal remnant is a congenital anomaly caused by a failure in the obliteration of the allantois into the median umbilical ligament.^1^ Failure of urachal obliteration can occur at different levels leading to a variety of clinical presentations. Urachal remnants can be asymptomatic or can present with a draining umbilicus or infected urachal cyst. Given the wide variety of symptoms, diagnosis and management are often delayed and varied between centers and providers.^2^

## 2 Case presentation

**Fig. 1:**
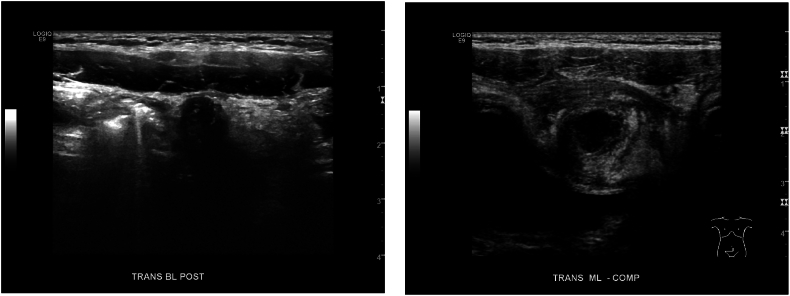
Pre-operative ultrasound.

**Fig. 2:**
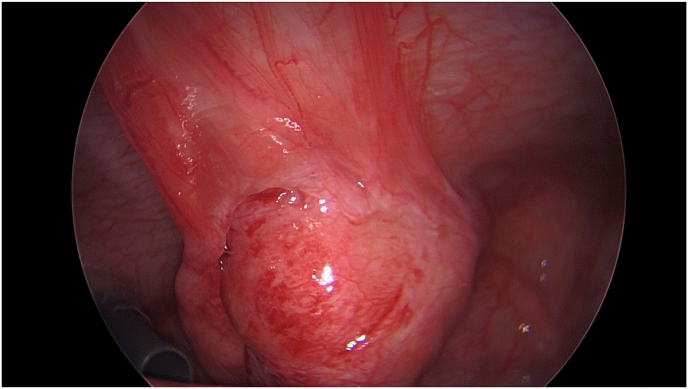
Laparoscopic view of urachal abscess.

**Fig. 3:**
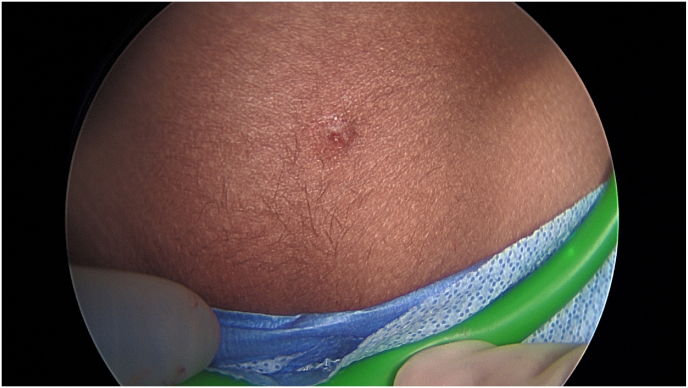
Suprapubic sinus tract.

A 4-year-old female patient presented to the emergency room with two days of abdominal pain and constipation without associated nausea, vomiting, or fevers. Ultrasound was performed and was interpreted as suspect appendicitis with noncompressible, enlarged, tubular structure (Fig. 1a–b). Urinalysis at that time showed moderate leukocyte esterase and 3+ bacteria per high power field with negative culture. She had a normal white blood cell count without left shift and unremarkable chemistries. On further interview, patient was noted to have a small wound or sinus in the suprapubic region. This had been present since birth and at time of presentation to emergency room, was noted to have new onset purulent drainage. Given concern for acute appendicitis, patient was taken to the operating room for diagnostic laparoscopy and appendectomy. Appendix appeared mildly inflamed and patient was noted to have a rounded, erythematous structure at the dome of the bladder concerning for infected urachal remnant (Fig. 2). A probe was inserted into the sinus tract (Fig. 3) but could not be clearly visualized by laparoscopy. The appendix was removed, and patient was kept on antibiotics for a total of 7 days post operatively.

**Fig. 4:**
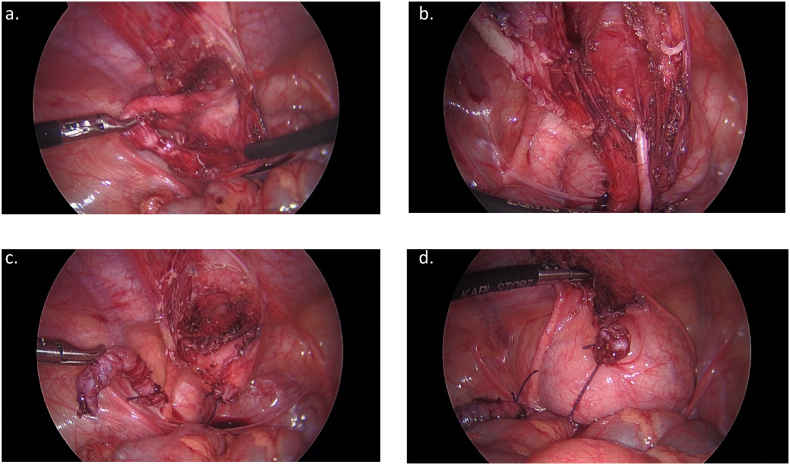
Intraoperative view of urachal remnant during laparoscopic excision.

A repeat ultrasound was performed approximately four weeks after surgery and demonstrated a linear tract extending from the bladder dome cranially consistent with a urachal remnant. Of note, this could not be followed to the umbilicus and there was no associated fluid collection. She was taken back to the OR seven weeks after initial operation and was found to have long tubular structure extending from the dome of the bladder to the suprapubic sinus, consistent with a urachal remnant (Fig. 4a). There was no extension towards the umbilicus and previously seen inflammation and abscess was resolved. Appendiceal stump was unremarkable. Procedure was performed using the same infraumbilical incision and right and left upper quadrant working ports. The urachal remnant was dissected using a combination of blunt and cautery dissection (Fig. 4b). Once isolated to the level of suprapubic sinus a lacrimal duct probe was inserted in attempt to cannulate the tract. This could only be passed to the level of the fascia consistent with a partially obliterated remnant and no ongoing drainage. The remnant was divided at the level of the abdominal wall and transected at the bladder using two absorbable braided endo-loops (Fig. 4c). The bladder was instilled with methylene blue and saline and there was no evidence leak (Fig. 4d). An elliptical incision was made around the suprapubic sinus tract, and this was excised to the level of the fascia. Foley catheter was removed post operatively and patient was discharged home the same day and has recovered without complication.

## 3 Discussion

Urachal anomalies arise from the embryologic descent of the bladder into the pelvis. In normal development, there is complete obliteration of the urachus to create a fibrous cord known postnatally as the median umbilical ligament. Urachal anomalies, or remnants, may have a range of presentations depending on the extent of patency of the urachus. These range from a completely patent urachus with urinary drainage from the umbilicus to an asymptomatic bladder diverticulum to a blind ending umbilical sinus or cyst. Urachal remnants were first described by Cabriolus in 1550.^3^ Since that time numerous case reports and series have reported on the variability in presentation and rarity of disorder associated with delayed or inaccurate diagnosis.^4^ Having a high index of suspicious is imperative to appropriate work up and diagnosis.

Based on limited epidemiologic data, urachal remnants are found in 1 % of pediatric patients who undergo imaging with an estimated incidence based on autopsy reports of 1 in 7610 for patent urachus and 1 in 5000 for urachal cysts.^5^ In patients who are symptomatic, the presentation usually consists of fluid drainage with possible infection. Less common presentations include a palpable mass, abdominal pain, urinary tract infection, urinary stones, bowel obstruction or perforation, fistula, bleeding, and malignancy. Malignant transformation, while rare, has been described in urachal remnants accounting for 0.1–0.3 % of all bladder malignancies and 20–39 % of bladder adenocarcinomas.^6^ Given the rarity of diagnosis, the number needed to treat to prevent the risk of urachal adenocarcinoma in adulthood exceeds 5700 prophylactic excisions. These numbers make prophylactic excision for malignancy alone less practical. When taking into account the risk of infection and other symptoms, however, excision has been generally recommended for those lesions that do not resolve spontaneously in the first two years of life.^7^

In the patient described, there were historical concerns of urachal remnant based on known history of suprapubic sinus but work up had been deferred for the first four years of life. There is a prior report of urachal remnant identified at the time of open appendectomy for appendicitis,^8^ with the subsequent use of laparoscopy increasing the visualization of the lower midline abdominal wall. The misdiagnosis of a urachal cyst for acute appendicitis has been described previously with increased incidence with the use of point of care ultrasound as primary mode of diagnosis.^9^ In the case report by Quinn et al. a pediatric patient presenting with abdominal pain and leukocytosis is diagnosed with suspected perforated appendicitis with periappendiceal abscess. They, like our patient, were started on antibiotics and taken for laparoscopic appendectomy. At time of surgery, they were similarly noted to have a minimally inflamed appendix with an inflammatory mass adjacent to the bladder. A primary excision was performed with a good outcome. Misdiagnosis of urachal remnant presenting with abscess is common and reported in up to 35–55 % of patients.^10^

Controversy exists in the literature between upfront resection, as done in the aforementioned case report, and staged excision of infected urachal remnants. For patients presenting with acute infection, many recommend a two-stage approach with antibiotics and possible drainage procedures prior to formal surgical excision. While the literature is largely limited to case reports and series, there is evidence to suggest decreased post operative complications such as wound infection and urine leak for a staged approach compared to immediate excision.^10^ One advantage of a staged approach with drainage is more rapid resolution of inflammatory processes resulting in an easier surgical excision. Drainage has been described percutaneously or open and may also provide a means for performing additional radiographic studies.^11^^,^^12^

In contrast to patients presenting with an abscess where management of infection, at a minimum, is required, there is ongoing debate about the management of the asymptomatic urachal remnant. As described, many advocate for surgical excision because of the risk of malignant transformation. Other proponents of surgical excision irrespective of presentation report surgery is primarily aimed at managing complications including infection, bladder outlet obstruction, or other complications related to the cyst itself.^1^ In contrast, Naiditch et al. describe a cohort of patients in whom spontaneous regression is observed.^13^ While the time frame for spontaneous resolution varies in the literature, asymptomatic or incidental diagnoses are more likely to be managed by a urologist than a general surgeon thus making conservative management more commonly performed by urologists. Studies describing the long-term outcome for conservative management, however, are limited in sample size and duration of follow up.^2^

## 4 Conclusion

Urachal remnants include a range of rare congenital anomalies with the majority being diagnosed incidentally. While the majority of patients present with umbilical drainage, a high index of suspicion is needed to identify patients with less common presentations, including a suprapubic sinus with abdominal abscess.

## CRediT authorship contribution statement

**Sarah Cairo:** Conceptualization, Data curation, Investigation, Methodology, Supervision, Writing – original draft, Writing – review & editing. **Gabriella Grisotti:** Investigation, Writing – original draft, Writing – review & editing. **Hanmin Lee:** Conceptualization, Investigation, Methodology, Writing – review & editing.

## Declaration of competing interest

The authors declare that they have no conflicts of interest.
